# Phenolic-Rich Extracts of *Galenia africana* and *Tulbaghia violacea* Accelerate Keratinocyte Migration and Mitigate Oxidative Stress to Enhance Wound Healing

**DOI:** 10.3390/plants14223523

**Published:** 2025-11-19

**Authors:** Banele Ndlovu, Randall Fisher, Farzana Fisher (née Rahiman)

**Affiliations:** 1 Skin Research Lab, Department of Medical Biosciences, University of the Western Cape, Cape Town 7535, South Africa; 3470783@myuwc.ac.za; 2 Radiation Biophysics Division, Separated Sector Cyclotron Lab., NRF iThemba LABS, Old Faure Rd, Eerste River, Cape Town 7100, South Africa; rfisher@tlabs.ac.za

**Keywords:** medicinal plants, wound healing, antioxidant activity, phenolic content

## Abstract

The rising prevalence of chronic burn wounds in South Africa places a significant burden on healthcare, driving the search for cost-effective, alternative treatments. Despite their long history of traditional use for skin ailments, the specific wound healing mechanisms of South African species *Galenia africana* L. and *Tulbaghia violacea* Harv. remain scientifically unvalidated, representing a critical knowledge gap and a novel area of inquiry. This study sought to evaluate the physiological and cellular effects of these plant extracts, specifically investigating their influence on keratinocyte function and oxidative stress. Initial analysis of crude ethanolic extracts confirmed the presence of key phenolic compounds like ferulic acid and rutin in both plants, with *G. africana* exhibiting a stronger overall radical-scavenging activity. In vitro assays were performed on the human keratinocyte (HaCaT) cell line. Both *G. africana* and *T. violacea* extracts significantly enhanced cell viability (at 40–80 μg/mL) and demonstrably reduced intracellular reactive oxygen species (ROS) levels, indicating a protective antioxidant effect. Critically, in the scratch wound assay, both extracts significantly accelerated cell migration and wound closure, achieving 76% (*G. africana*) and 88% (*T. violacea*) closure within 24 h. These findings suggest that *G. africana* and *T. violacea* extracts act to support keratinocyte proliferation and migration while simultaneously mitigating oxidative cellular damage. This work provides an important mechanistic basis supporting the traditional use of these specific, regionally important plants and highlights their high therapeutic potential as novel, cost-effective agents to accelerate skin repair and recovery.

## 1. Introduction

A wound is defined as a disruption to the skin’s integrity, characterised by damage not only to the epidermis and dermis, but also extending to the underlying subcutaneous tissue, including bone and muscle [1]. Acute superficial abrasions are wounds that typically heal within a predictable timeframe, while chronic wounds fail to progress through normal healing stages and often require medical intervention [2]. Chronic wounds present a significant challenge to healthcare systems, particularly in South Africa (SA), where there is a high prevalence of burn wounds resulting from urban and rural fires [3]. Wildfires are a frequent occurrence in SA, often exacerbated by informal housing structures constructed from highly flammable materials such as wood and plastics [4]. In addition, the social and economic conditions in certain communities, such as overcrowding and limited access to fire prevention resources, contribute to the high incidence of burn injuries. The increasing costs of dermatological care and medication make hospital treatments unaffordable for many, leading them to seek alternative treatment options or delayed treatment [5]. Consequently, a delay in medical attention often results in severe infections, which can lead to conditions such as hypovolemia and septicaemia [6]. Therefore, there is an urgent need to explore alternative, cost-effective treatment options that promote faster healing.

Traditional and complementary medicine remains an essential resource, particularly in Africa, where it contributes substantially to primary healthcare services [7]. Li et al. [8] reported that the use of leaves as a wound dressing was one of the earliest forms of treatment to prevent infection, reduce pain and increase the rate of wound closure. In SA, approximately 27 million people use herbal medicine, with a significant portion of these remedies prescribed by traditional practitioners [9]. The Western Cape (WC) province has over 8650 medicinal plant species, 65% of which are endemic to the region [10]. Ethnobotanical studies have reported that traditional herbal practitioners in the WC region regularly incorporate these medicinal plants into their healing practices [11].

Some of the medicinal plants used by WC herbal practitioners include *Aizoon africanum* (L.) *Klak* (*syn. Galenia africana* L.) and *Tulbaghia violacea* Harv. [12,13]. *G. africana* L. is commonly known as “kraalbos”, belongs to the Aizoaceae family and is found in the WC, Eastern Cape, Northern Cape and Free State provinces [12,14]. Traditional healers have topically applied different pastes, decoctions and lotions from *G. africana* to treat wounds [15,16]. The ethanolic extracts of *G. africana* have demonstrated selective cytotoxicity in cancerous cells by significantly reducing their viability while exerting minimal effects on normal cells [16,17,18]. *T. violacea*, or “wild garlic”, belongs to the Amaryllidaceae family and is widely distributed in the WC, Eastern Cape and KwaZulu-Natal; it has significant medicinal and culinary applications [19,20]. Traditionally, *T. violacea* is used to flavour food and to treat a range of ailments, including fever, colds, hypertension and stomach problems [21]. This plant emits a strong garlic-like odour and can be crushed on the skin as an insect repellent without causing irritation [19]. Steroidal saponins isolated from *T. violacea* have demonstrated dermatological effects, including anti-inflammatory and antioxidant properties, which support its traditional use in wound care products [22,23].

Although ethnobotanical studies have documented the traditional cosmetic applications of *G. africana* and *T. violacea,* these reports mostly rely on anecdotal evidence, and there is limited scientific information available on their wound healing properties [24,25,26]. More rigorous scientific investigations are required to substantiate the therapeutic efficacy and elucidate the mechanisms of action of these plants as wound healing agents. Therefore, this study aimed to evaluate the wound healing and antioxidant effects of *G. africana* and *T. violacea* as potential alternative therapies.

## 2. Materials and Methods

### 2.1. Materials and Reagents

Fluorescein sodium salt (Cat no. F6377), AAPH (2,2-Azobis (2-methylpropionamidine) dihydrochloride (Cat no: 440914), Perchloric acid, Trolox (6-Hydrox-2,5,7,8-tetramethylchroman-2-carboxylic acid) (Cat no. 238831), TPTZ (2,4,6-tri [2-pyridyl]-s-triazine) (Cat no. T1253), L-Ascorbic acid (Cat no: A5960), Iron (III) chloride hexahydrate, di-sodium hydrogen orthophosphate dehydrate (Na_2_HPO_4_·2H_2_O) (Cat no. 5822880EM), Folin–Ciocalteus phenol reagent (Cat no. 109001), Sodium Carbonate (Cat no. 223530), gallic acid (Cat no. G7384), rutin (Cat no. PHL89270), chlorogenic acid (Cat no. C3878), Coumaric acid (Cat no. C9008), caffeic acid (Cat no: C0625), ferulic acid (Cat no: PHR1791), quercetin (Cat no. Q4951), gallic acid (Cat no. 27645), catechin (Cat no. C1251), epicatechin (Cat no. E1753) and protocatechuic acid (Cat no. 03930590) were obtained from Sigma-Aldrich (St. Louis, Mo, USA). Acetate Buffer (Cat no. 1021000), Hydrochloric acid (Cat no: 100319 LP), Hexane (Cat no. 2868040 LC), Acetone (Cat no: 1022040 LC), were purchased from Saarchem Laboratory Chemicals.

### 2.2. Plant Material and Extract Preparation

Commercially prepared ethanolic extracts of *G. africana* and *T. violacea* ethanolic extracts were purchased from Brenn-O-Kem (PTY) Ltd. (Wolseley, South Africa) and Zuplex Botanicals (Pty) Ltd. (Amanzimtoti, South Africa), respectively. The suppliers provided the following specifications and preparation methods for each extract:

*G. africana* extract: The plant material (Batch no: IBTKB01/2023) was harvested in Komaggas, Namaqua District, Northern Cape Province and identified at the Herbarium, Department of Botany, University of the Western Cape, Bellville, by Mr F. Weitz (Vries 1; herb no 6595). The leaves and shoots of *G. africana* were air-dried, ground and macerated in 60% ethanol at a 20% (*w*/*v*) ratio for 12 h at room temperature. The resulting 60% ethanolic extract was supplied as a stock solution with a concentration of 20 mg/mL.

*T. violacea* extract: The plant material (Batch no: UWCTVT-09/24) was cultivated by Muthi Futhi at its site in Dakeni, KwaZulu-Natal. The supplier confirmed the plant’s taxonomic identity. The whole plant, including aerial parts and bulbs, was chopped, sun-dried and ground into a fine powder using a hammer mill. The extract was prepared by macerating 100 g of the powder in 50% ethanol (Alco NCP 96.4%, Durban, South Africa) at room temperature for 48 h, followed by filtration through a 25 μm filter. The extract was supplied as a 100 mg/mL solution, representing a 10% (*w*/*v*) preparation.

For the phytochemical and antioxidant assays, the extracts were diluted to a final concentration of 1 mg/mL, consistent with a previous study [27]. For the cell culture experiments, the extracts were further diluted to 0.1 mg/mL, a concentration considered physiologically relevant [28].

### 2.3. Phytochemical Analysis

#### 2.3.1. Total Polyphenolic Content

The total polyphenolic content (TPC) of *G. africana* and *T. violacea* ethanolic extracts were determined spectrophotometrically using the Folin–Ciocalteu method Siddiqui et al. [29]. Briefly, 25 μL of the samples and standards were mixed with 125 μL of the Folin–Ciocalteu reagent (10%) in a 96-well plate and left to stand for 5 min. Sodium carbonate solution at 100 μL (20% *w*/*v*) was then mixed with the samples, incubated for 2 h at room temperature and then read at 765 nm. Gallic acid (800 mg/mL in 10% ethanol) was used to set up the standard curve, and the total polyphenolic content of the samples was expressed as gallic acid equivalents (GAEs) in mg per gram dry weight. All the samples were analysed in triplicate.

#### 2.3.2. Determination of Total Flavonoid Content

The flavanol content was determined using the method previously described Tyavambiza et al. [27], with minor modification. Briefly, 50 μL of the *G. africana* and *T. violacea* extracts (1 mg/mL) were mixed with 250 μL of a 50 g/L 4-Dimethylamino-cinnamaldehyde in a 96-well plate and incubated for 30 min at room temperature. The absorbance readings were read at 640 nm. Results were expressed as milligram catechin equivalent per gram (mg CE/g). The flavonol content was determined according to the method in [30] where 12.5 μL of 0.1% HCl in 95% ethanol and 225 μL of 2% Hydrochloric acid was added to 12.5 μL of the *G. africana* and *T. violacea* extracts (1 mg/mL), incubated for 30 min at room temperature. The absorbance was read at 360 nm. Quercetin (1.0 mM) was used as a standard and results were expressed as milligram quercetin equivalent per gram (mg QE/g). Total flavonoid content was determined as the sum of the quantified flavanol and flavonol fractions.

### 2.4. Quantitative Analysis of Extracts by High-Performance Liquid Chromatography (HPLC)

High-performance liquid chromatography (HPLC) has been used to isolate phytochemicals from plant extracts to purify, identify and quantify individual compounds [31]. These identified compounds may possess therapeutic properties, including antioxidant and anti-inflammatory activities [32]. HPLC analysis was performed using an Agilent 1200 series HPLC system (Santa Clara, CA, USA) with a UV detector. The compound separation and analysis were conducted using an Agilent 1200 Series Quaternary Pump (Cat. No G1311A, Agilent Technologies, Santa Clara, CA, USA) column: Phenomenex Luna 5-micron C18, 150 × 4.6 mm. The flow rate was 1 mL/min, and the injection volume was 20 μL. The mobile phase consisted of A: water containing 0.1% acetic acid and B: methanol containing 0.1% acetic acid. For equilibration, the gradient conditions included time 0 min = 95% A to time 35 min = 80% B, with detection at wavelengths of 280, 320 and 350 nm. Quantification was performed using external calibration curves. The commercially available standards (>99%), namely, rutin, quercetin, kaempferol, gallic acid, catechin, epicatechin, protocatechuic acid, ferulic acid, caffeic acid, coumaric acid and chlorogenic acid, were used. The compounds in *G. africana* and *T. violacea* were identified by comparing their retention times with those of the standards. Individual stock solutions and the calibration curves were constructed from five serial dilutions in the range of 1–50 mg/L, all showing excellent linearity (R^2^ > 0.999). All analyses were performed in triplicate.

### 2.5. Antioxidant Studies

The antioxidant activity of the *G. africana* and *T. violacea* ethanolic extracts were determined using common colorimetric assays, namely, DPPH, FRAP and ORAC.

#### 2.5.1. DPPH Radical Scavenging Assay

The antioxidant activity of *G. africana* and *T. violacea* ethanolic extracts were evaluated using the DPPH (2,2-diphenyl-1-picrylhydrazyl) (Sigma-Aldrich, Darmstadt, Germany; M.W. 394.32) assay. DPPH is a free radical with a deep violet colour that loses its colour when it is reduced in the presence of an antioxidant molecule [33]. Therefore, the more antioxidants are present, the more colour the sample loses. The DPPH activity was measured using the method adapted from Chaves et al. [34]. Briefly, the solution of the radical was prepared by dissolving 2.4 mg DPPH in 100 mL of methanol. 0.1 mL of the samples was added to 3.9 mL of DPPH solution in a 96-well plate (Greiner Bio-One, Kremsmunster, Austria), then incubated in the dark for 30 min at room temperature. The absorbance was read at 515 nm using a SpectraMax i3X multi-well reader (Molecular Devices, San Jose, CA, USA). Trolox (1.0 mM in ethanol) was used as a standard and a calibration curve was plotted with % DPPH scavenged versus concentration of Trolox. The experiment was carried out in triplicate. The absorbance of the samples was compared to a standard curve and the results were expressed as mol TE/g. Free radical scavenging activity was calculated using the following equation [35]:

% DPPH radical-scavenging = [(Absorbance of control − Absorbance of test Sample)/(Absorbance Of control)] × 100

#### 2.5.2. Oxygen Radical Absorbance Capacity Assay

The total antioxidant capacity of *G. africana* and *T. violacea* ethanolic extracts were evaluated using Oxygen Radical Absorbance Capacity (ORAC). The assay evaluates the radical-scavenging activity of compounds against peroxyl radicals generated by 2,2′-azobis(2-amidino-propane) dihydrochloride (AAPH) using a fluorescent protein as oxidation probe [36]. The assay was adapted from López et al. [37], briefly, 12 μL of the extract samples were mixed with 138 μL of the fluorescein (μM), which served as the target for free radicals in a 96-well plate. 50 μL of AAPH (768 μM) was added to the plate and fluorescence (emission 538 nm, excitation 485 nm) was analysed every 1 min for 2 h using a Fluorescence plate reader. Trolox (1.0 mM in ethanol) was used as a standard. The absorbance of the samples was compared to a standard curve, and the results were expressed as mol TE/g.

#### 2.5.3. Ferric Reducing Antioxidant Power Assay (FRAP) Assay

The colorimetric Ferric Reducing Antioxidant Power (FRAP) assay was used to measure the reduction of ferric ion (Fe^3+^) complex by the extract according to the method performed by Gohari et al. [38]. The assay is based on the reduction of Fe^3+^ 2,4,6-Tri(2-pyridyl)-s-triazine (TPTZ) complex (colourless complex) to Fe^2+^-tripyridyltriazine (blue-coloured complex) formed by the action of electron-donating antioxidants at low pH. The FRAP reagent was prepared by mixing 10 mL of 300 mM acetate buffer, 10 mL TPTZ in 40 mM HCl and 20 mM FeCl_3_.6H_2_O. The working solutions of the FRAP reagent (1–5 mL) were mixed with the sample (*G. africana* and *T. violacea*) in a 96-well plate to a final concentration of 20 μL and incubated at room temperature for 30 min in the dark. The assay was detected by the reduction of Fe^3+^ TPTZ to ferrous (Fe^2+^) in the presence of an electron-donating antioxidant. The plate was read at 593 nm. Ascorbic acid (1.0 mM in distilled water) was used in the preparation of the standard solutions. The absorbance of the samples was compared to a standard curve, and the values were expressed as mol AAE/g.

### 2.6. Cell Culture Studies

To investigate the wound healing potential of *G. africana* and *T. violacea* ethanolic extracts in vitro, the cell viability, scratch and intracellular ROS assays were conducted.

#### 2.6.1. Cell Line

The immortalised human keratinocytes (HaCaT) cell line was purchased from Cell Line Service GmbH (Cat no. 300493, Eppelheim, Germany). For the experiments, the ethanolic extracts were diluted in to 0.1 mg/mL Dulbecco’s Modified Eagle’s Medium (DMEM) (Cat no. BE12-709F, Lonza, Cape Town, South Africa) supplemented with 5% heat-inactivated foetal bovine serum (HI-FBS, Hyclone, Little Chalfont, UK) and 1% of antibiotics such as 250 μg/mL amphotericin B (Cat no. 15290018, GIBCO BRL, Grand Island, NY, USA), penicillin 10,000 Units/mL and streptomycin 10,000 μg/mL (Cat no. 15140122, GIBCO BRL, Grand Island, NY, USA). The treatment concentrations for the extracts were 20, 40, 60, 80, and 100 μg/mL.

#### 2.6.2. Cell Viability and Proliferation

The effects of the plant extracts on HaCaT cells were evaluated using a colorimetric cell viability assay to quantify viable cell number in multi-well microplates, with minor modifications to a previously described protocol [39]. Cell viability was determined using 3-(4, 5-Dimethylthiazol-2-yl)-2, 5-Diphenyltetrazolium (MTT) (Bromide, Sigma Aldrich, St. Louis, MI, USA), a positively charged tetrazolium salt that readily penetrates viable cells and is reduced to an insoluble, coloured formazan. This assay was also used to determine the optimal concentrations used to treat the cells for wound healing. Briefly, 5 × 10^3^ cells/well HaCaT cells were seeded into 96-well tissue culture plates (Greiner Bio-one, Kremsmunster, Austria) and allowed to adhere overnight. Subsequently, the cells were treated with 20, 40, 60, 80, and 100 μg/mL concentrations of the plant extracts and incubated for 24 and 48 h. Following incubation, 10 μL of MTT solution (5 mg/mL) was added to each well and the cells were incubated at 37 °C for 3 h. Dimethyl sulfoxide (DMSO) (Cat. no D2650, Sigma, Deutschland GmbH, Schnelldorf, Germany) was used to solubilize the formazan product and absorbance was measured at 570 nm using a microplate reader. Cells without extract treatments served as a negative control, while cells treated with 1% Triton-X 100 were used as a positive control.

#### 2.6.3. Scratch Assay

A scratch wound assay was used to assess the migration rate of HaCaT cells following treatment with *G. africana* and *T. violacea*. Cells were seeded in serum-free media at a density of 2.5 × 10^5^ cells/well in Culture-Inserts (Cat. 81176, ibidi GmbH, Gräfelfing, Germany) inside 24-well plates (Cat. no. 662160, Greiner Bio-one, Kremsmunster, Austria) and allowed to adhere. The cells were then treated with serum-free culture media containing the optimum concentrations of the extracts for 12 h. After incubation, the ibidi inserts were removed to create an artificial linear wound with a 500 µm diameter. Images of each wound were captured at 0, 4, 8, 12, 16, 20 and 24 h using an inverted microscope (Zeiss PrimoVert, Carl Zeiss Microscopy GmbH, Jena, Germany). To measure the percentage area of cell migration and gap closure, ImageJ software version 1.8.0 (National Institutes of Health (NIH), Bethesda, MD, USA) was used for analysis and the area of the scratch wound was quantified using the MiToBo plug-in for ImageJ [40].

#### 2.6.4. Dichlorofluorescin Diacetate (DCFDA) Cellular Antioxidant Detection Assay

Intracellular reactive oxygen species (ROS) levels are integral to the wound healing processes by regulating cellular responses; however, due to excess ROS levels, inflammation can cause tissue damage and impede proper healing [41]. Cellular ROS production was determined using the DCFDA (2′,7′-dichlorodihydrofluorescein diacetate) probe as an indicator of ROS generation after pre-treated HaCaT cells were exposed to tert-butyl hydroperoxide (tBHP) an exogenous inducer of oxidative stress [42]. DCF-DA is a non-fluorescent compound, taken up by cells and deacetylated by intracellular esterases. ROS then oxidises the resulting deacetylated product to 2′,7′-dichlorofluorescein, a fluorescent product [43]. Following the protocols previously described [44,45] with minor modifications, HaCaT cells were seeded at a density of 5 × 10^3^ cells/well in 96-well black plates (Greiner Bio-One, Kremsmunster, Austria) and allowed to adhere overnight. The cells were then treated with the different concentrations of the extracts and incubated for 24 and 48 h. Following incubation, the cells were washed with Hanks’ Balanced Salt Solution (HBSS) (Cat. 14025050, Gibco, Waltham, MA, USA) and exposed to 200 µM tBHP (458139, Sigma-Aldrich, St. Louis, MO, USA) solution and incubated for 1 h. The cells were then loaded with 10 µM DCF-DA probe in HBSS and incubated for 30 min in the dark at 37 °C. The Fluorescence of the cells was then measured using a microplate reader (Glomax Multi Detection System, Promega, WI, USA) (λex = 495 nm/λem = 525 nm). Cells, treated with 200 µM tBHP only were used as a positive control. To analyse ROS levels, the fluorescence values for all treatment groups, including the positive control were normalised to the negative control (untreated cells). All subsequent statistical analysis was conducted using these values.

### 2.7. Data Analysis

Results were reported as mean ± standard error of the mean (SEM) of three independent variables. GraphPad Prism version 8.0.1 (GraphPad Software, Inc., San Diego, CA, USA) was used to assess the statistical significance and differences among groups, which were evaluated using one-way analysis of variance (ANOVA). Tukey’s test was performed for the comparison of means for the corresponding results. Differences between values with a *p* < 0.05 were considered statistically significant.

## 3. Results and Discussion

### 3.1. Phytochemical Analysis and Antioxidant Activity

Table 1 shows that *G. africana* displayed significantly higher (*p* < 0.05) polyphenolic content (106.39 ± 3.51 mg GAE/g) compared to *T. violacea* (78.57 ± 4.16 mg GAE/g), representing 13.3% and 9.8% of the gallic acid standard (800 mg GAE/g), respectively. *G. africana* contained (39.79 ± 0.27 mg QE/g) flavonoids, corresponding to 4% of the quercetin standard, while *T. violacea* showed no detectable flavonoid content. According to Piluzza and Bullitta [46], the total polyphenolic content generally correlates with antioxidant assays such as DPPH and FRAP, suggesting that phenolic compounds contribute to antioxidant activity. Correspondingly, the DPPH assay revealed that both extracts demonstrated substantial radical scavenging activity, with *G. africana* found to be significantly higher (187.42 ± 11.09 µmol TE/g; *p* < 0.05) than *T. violacea* (117.60 ± 10.97 µmol TE/g), representing 18.7% and 11.8% of Trolox standard activity, respectively. These results are consistent with similar findings reported in a previous study on *Alkanna corcyrensis* extracts, which showed high scavenging ability (227.01 ± 2.15) [47]. In the FRAP, *G. africana* (195.91 ± 7.38 µmol AAE/g) showed significantly (*p* < 0.05) higher ferric reducing activity than *T. violacea* (116.25 ± 21.77 µmol AAE/g), equivalent to 19.6% and 11.6% of the ascorbic acid standard’s activity, respectively. The ORAC assay further corroborated these findings, with *G. africana* demonstrating a notably higher antioxidant capacity (1247.83 ± 21.70 µmol TE/g; *p* < 0.05) compared to *T. violacea* (172.82 ± 21.47 µmol TE/g). Most notably, *G. africana*’s ORAC activity exceeded the Trolox standard by 25%, while *T. violacea* showed only 17.3% of the standard’s activity. This suggests that *G. africana* possesses stronger peroxyl radical scavenging activity relative to *T. violacea*. A similar study reported that *Tulbaghia* species had relatively low antioxidant capacity, suggesting that their bioactivities may rely on individual compounds being more effective when isolated in pure form [48]. These results show that *G. africana* has stronger antioxidant potential, likely due to its higher polyphenolic content compared to *T. violacea*. Previous studies have demonstrated a correlation between phenolic compounds and antioxidant activity, as phenolics are effective reducing agents and free radical scavengers, which significantly contribute to antioxidant capacity [27].

### 3.2. HPLC Chromatographic Profiles of the Plant Extracts

**Figure 1:**
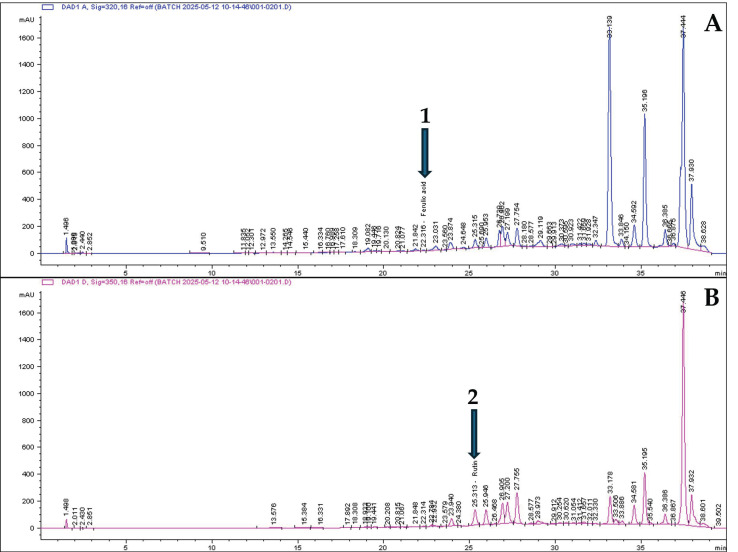
HPLC chromatograms of the bioactive compounds from *G. africana* ethanolic extracts at wavelengths of (**A**): 320 and (**B**): 350 nm. Peaks indicated by arrows and numbers represent 1: Ferulic acid and 2: Rutin identified by comparison with commercially available standards. Chromatographic profiles: phenolic acids monitored at 320 nm (blue line) and flavonols monitored at 360 nm (pink line). The unlabelled peaks represent other components of the complex extract that were not identified in this targeted analysis.

**Figure 2:**
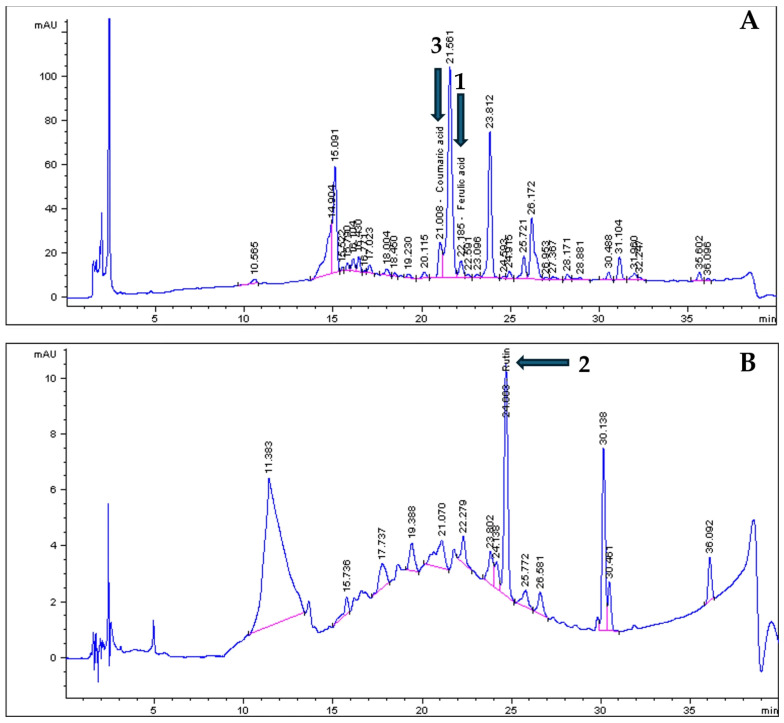
HPLC chromatograms of the bioactive compounds from *T. violacea* ethanolic extracts at wavelengths of (**A**): 320 and (**B**): 350 nm. Peaks indicated by arrows and numbers represent 1: Ferulic acid, 2: Rutin and 3: Coumaric acid identified by comparison with authentic standards. Chromatographic profiles: phenolic acids monitored at 320 nm (blue line) and flavonols monitored at 360 nm (pink line). The unlabelled peaks represent other components of the complex extract that were not identified in this targeted analysis.

The ethanolic extracts were subjected to HPLC analysis to further identify and quantify the phytochemicals present that contribute to the antioxidant properties of the plant extract (Table 2). Figure 1 displays the chromatograms of compounds identified from *G. africana* including ferulic acid (1.88 mg/mL) (Figure 1A) and rutin (35.87) (Figure 1B). Figure 2 shows compounds identified from the chromatograms of *T. violacea* such as ferulic acid (1.88 mg/mL), coumaric acid (13.58 mg/mL) (Figure 2A) and rutin (1.18 mg/mL) (Figure 2B). Various studies have widely reported on the use of these compounds in treatments for dermatological conditions [49,50,51]. Literature has shown that compounds such as rutin, coumaric acid and ferulic acid had strong free radical scavenging activity, phenolic content and are potent antioxidants [52,53,54]. Studies have found that ferulic acid and rutin possess antioxidant activity, are well absorbed by the skin and can effectively protect the skin from UVB radiation [50,51]. According to Choi et al. [55], rutin inhibits collagenase, an enzyme that degrades collagen, which plays a role in maintaining skin elasticity and reducing wrinkles. Reports on the natural phenolic compounds, such as coumaric acid, found that it can be used in skincare and wound healing due to its ability to reduce oxidative stress and mediate inflammation [49]. These results suggest that the phenolic compounds present in these plants could be associated with their traditional use for skin conditions such as wound healing and skin infections [56,57].

#### 3.2.1. The Effects of Ethanolic Extracts on Cell Viability in HaCaT Cells

**Figure 3:**
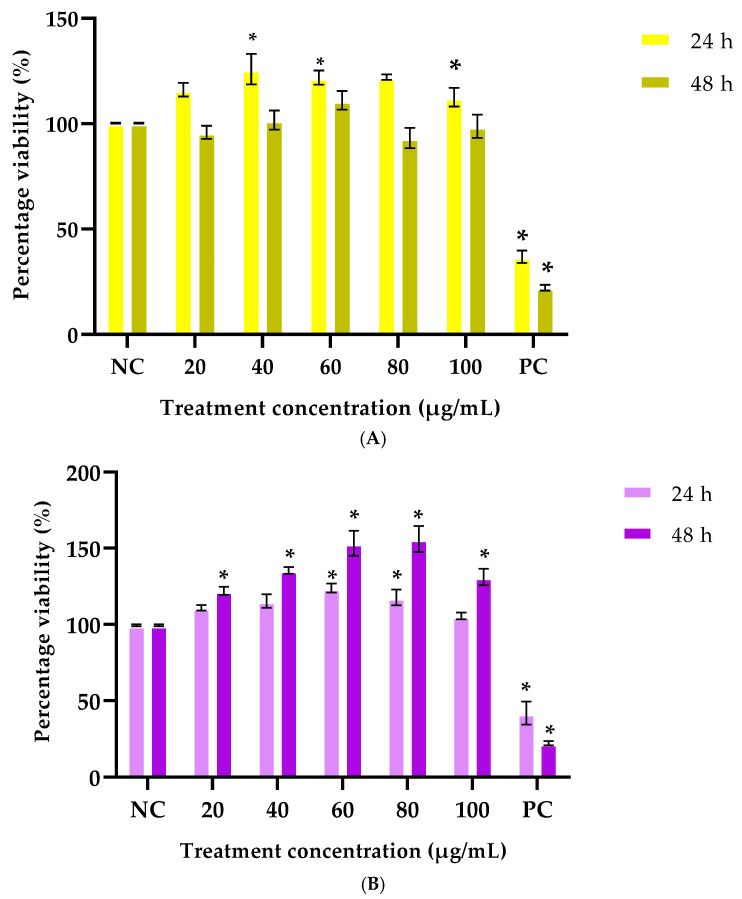
Cell viability and proliferation of normal skin (HaCaT) cells treated with 20, 40, 60, 80 and 100 µg/mL concentrations of *Galenia africana* (**A**) and *Tulbaghia violacea* (**B**) ethanolic extracts for 24 and 48 h were determined using an MTT assay. NC: negative control—untreated cells with media, PC: positive control- cells treated with 1% Triton-X 100. Data represent the mean ± SEM, n = 3, where * indicates statistical significance (*p* < 0.05) compared to the negative control.

To assess the effect of *G. africana* and *T. violacea* on cell viability, HaCaT cells were exposed to the ethanolic extracts at concentrations ranging from 20 to 100 μg/mL. Figure 3 shows that neither extract had an adverse effect on the viability of cells within these concentration ranges. However, a significant increase (*p* < 0.05) in cell proliferation was noted at concentrations between 40–80 μg/mL after 24 h of exposure to the *G. africana* extract, compared to the negative control (Figure 3A). The optimum concentration of *G. africana* for promoting cell proliferation was determined at 40 μg/mL as a significant 25% increase (*p* < 0.05) in cell growth after 24 h, compared to the negative control. A similar study on the toxicity potential of *G. africana* in human fibroblast cells reported that *Galenia*-gold nanoparticles did not significantly reduce cell viability after 24 h [58].

*T. violacea* induced a significant increase in cell proliferation at concentrations between 40–60 μg/mL after 24 h of exposure and between 20–100 μg/mL after 48 h compared to the negative control (Figure 3B). The optimum concentration of *T. violacea* for enhancing cell proliferation was determined to be 60 μg/mL after 24 h of exposure, due to a significant increase (*p* < 0.05) in cell viability compared to the negative control. Studies have shown that rutin, a compound isolated from *T. violacea*, demonstrated a concentration-dependent efficacy on HaCaT cell viability, exhibiting non-cytotoxic effects [59,60]. The cell viability results suggest that *G. africana* and *T. violacea* extracts could promote the cell proliferation phase of wound healing, leading to accelerated wound closure [61,62].

#### 3.2.2. The Effect of Ethanolic Extracts on Cell Migration

**Figure 4:**
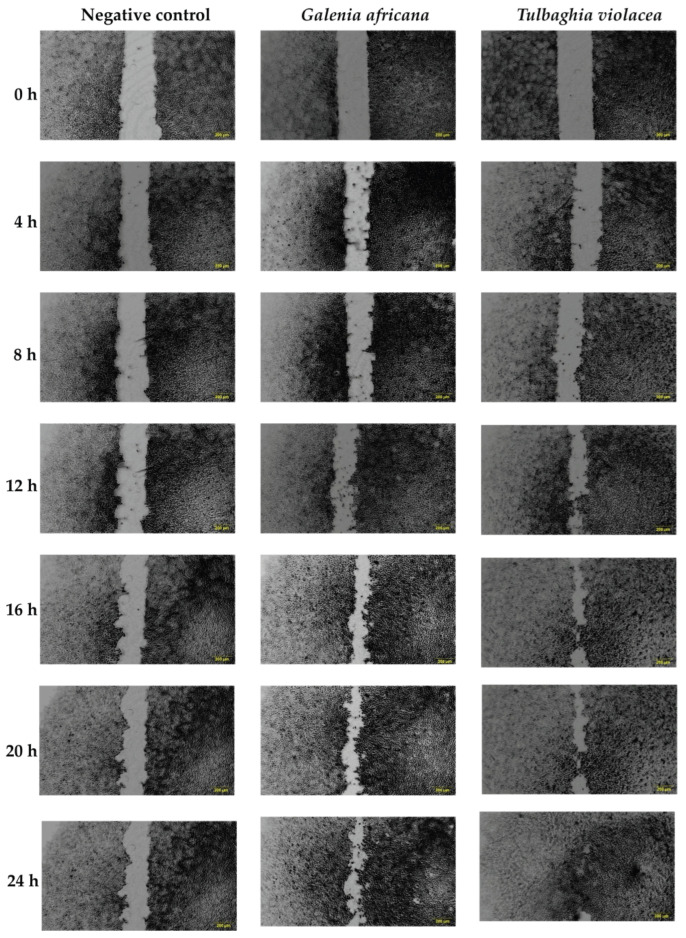
HaCaT cell migration percentage was determined using a scratch assay. Images show the wound area at 0 h (initial scratch) and the progression of wound closure at 4, 8, 12, 16, 20 and 24 h after treatment with the optimum concentration of 40 μg/mL for *Galenia africana* and 60 μg/mL *Tulbaghia violacea*. The negative control shows untreated cells with serum-free media. Scale bar: 200 µm.

In response to a disruption caused to the skin barrier, keratinocyte epidermal cells secrete cytokines such as interleukin 1 (IL-1) and growth factors, namely, vascular endothelial growth factor (VEGF) and platelet-derived growth factor (PDGF), which induce endothelial cell migration and angiogenesis in the wound bed [63]. Understanding the cell migration process is therefore an essential part of wound healing and was studied using a scratch wound assay (Figure 4). The negative control showed a slower gap closure compared to the extract-treated cells. After 24 h of exposure to the optimum concentrations of *G. africana* (40 μg/mL) and *T. violacea* (60 μg/mL), increased wound closure was observed. Both plant extracts promoted cell migration, with *T. violacea* achieving an 88% reduction in wound gap after 24 h, while *G. africana* had a 76% gap closure at 24 h compared to the initial scratch (baseline, 0 h). The accelerated wound healing effects observed may be associated with the modulation of signalling pathways that contribute to cell proliferation, migration and extracellular matrix remodelling. Previous studies have reported that rutin, ferulic acid and coumaric acid promote healing by modulating key factors crucial for tissue regeneration, including the upregulation of VEGF and collagen, while also influencing the activity of TGF-β3 pathway to regulate inflammation [52,64,65]. A study by Ghaisas et al. [65] reported that ferulic acid could inhibit lipid peroxidation, resulting in the restoration of normal VEGF levels, thereby promoting wound healing. The oral consumption and topical application of ferulic acid significantly increased hydroxyproline levels, an important amino acid used as a marker for collagen synthesis and repairing damaged skin cells [64]. Additionally, compounds such as coumaric acid and rutin have demonstrated wound healing properties by decreasing the duration of the inflammatory phase of wound healing while promoting angiogenesis, reducing oxidative stress and enhancing epidermal regeneration [59,66].

**Figure 5:**
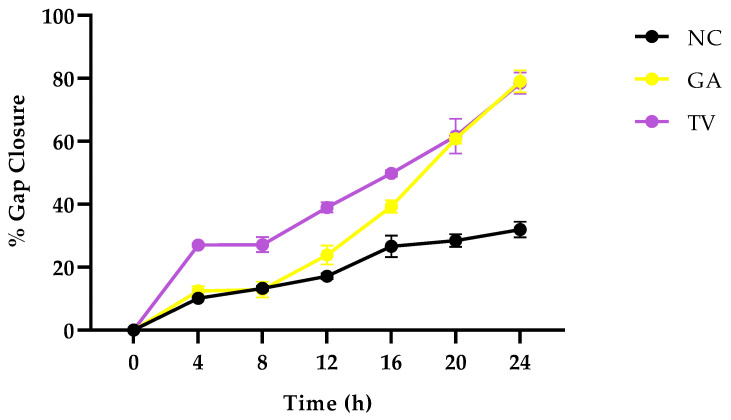
Percentage of wound gap closure over time in HaCaT cells pretreated with *Galenia africana* (GA) (40 μg/mL) and *Tulbaghia violacea* (TV) (60 μg/mL). NC: negative control—untreated cells with serum-free media. Data represent the mean percentage of the original wound area that has closed at each time point over the 24 h.

Figure 5 represents the percentage of wound closure over 24 h varied significantly between treatments. *T. violacea* promoted the most rapid healing, while *G. africana* resulted in a moderate speed but notably faster closure compared to the negative control, which showed a minimal change throughout the observed period. These results indicate that both plants effectively promote wound closure in vitro, which could be attributed to their ability to suppress inflammatory cytokines, resulting in a rapid progression from the inflammatory phase to the later stages of wound healing [67]. Previous studies have reported that compounds found in these plants, such as rutin, ferulic acid and coumaric acid, possess anti-inflammatory effects that suppress proinflammatory cytokines such as IL-6 and Tumor Necrosis Factor alpha (TNF-α), promoting angiogenesis and tissue regeneration [68,69,70]. *T. violacea* and *G. africana* could potentially increase the production of anti-inflammatory cytokines to modulate the inflammatory response, promoting rapid cell migration; however, further molecular experiments are required to corroborate this.

#### 3.2.3. Cellular Antioxidant Assay (CAA)

**Figure 6:**
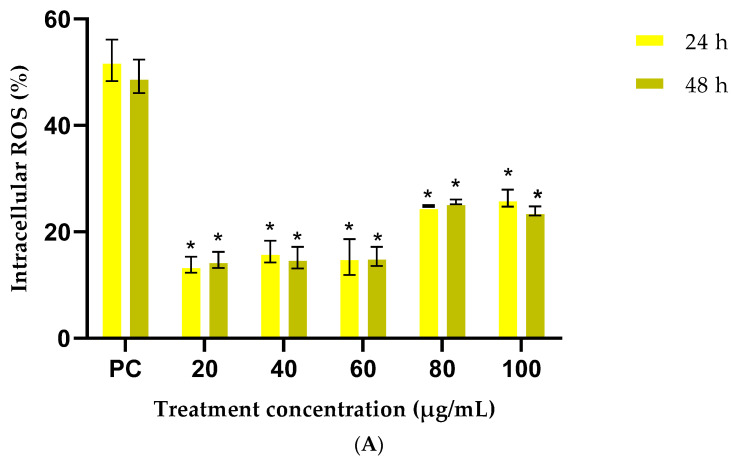

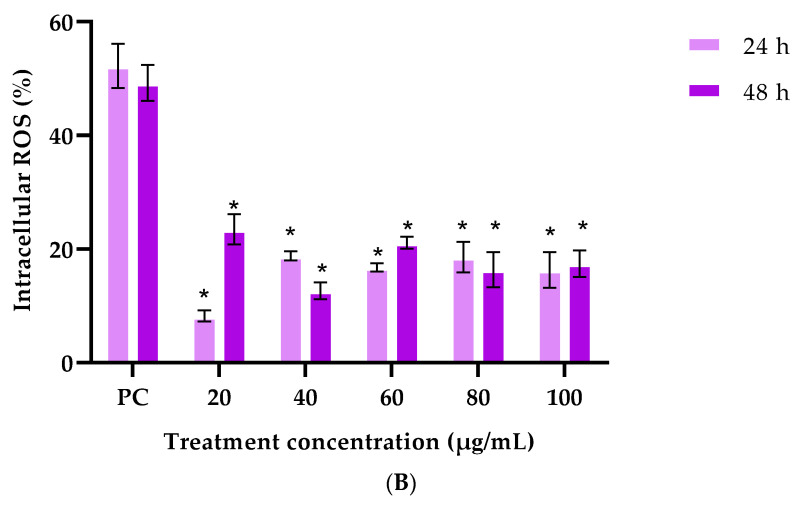
The effects of *Galenia africana* (**A**) and *Tulbaghia violacea* (**B**) on intracellular ROS formation in HaCaT cells exposed to the pro-oxidative agent tBHP for 24 and 48 h. Data represents normalised ROS production, expressed relative to the negative control (untreated cells). Cells treated with tBHP were used as a positive control (PC). Data are shown as mean ± SEM, n = 3, where * indicates statistical significance (*p* < 0.05) compared to the PC.

Oxidative stress is a major contributing factor to delayed wound healing, since it causes an increase in ROS production that adversely affects cellular lipids, proteins and DNA, leading to cellular and tissue dysfunction [71]. For the intracellular ROS assay, cells exposed to the positive control showed a significant increase in ROS activity, indicating the assay’s sensitivity and responsiveness for detecting intracellular ROS (Figure 6). Results show that cells stimulated with tBHP and pre-treated with different concentrations (20–100 μg/mL) of *G. africana* (Figure 6A) showed a significant decrease (*p* < 0.05) in intracellular ROS levels compared to the positive control. These results suggest that *G. africana* could potentially penetrate cells and act with strong radical scavenging potency in a stressed environment [72]. Several studies have corroborated the antioxidant effects of *G. africana,* with reports that it is effective in mitigating oxidative stress-related conditions such as cancer [17,18].

Stimulated tBHP cells pretreated with *T. violacea* at concentrations between 20–100 μg/mL (Figure 6B) had a significant reduction (*p* < 0.05) in ROS activity across all exposure times compared to the positive control. According to Afzal et al. [73], as part of their metabolic processes, healthy cells can maintain a certain baseline level of ROS. These results suggest that *T. violacea* could prevent the increase in ROS while also actively maintaining baseline ROS levels, suggesting that it can enhance the cells’ antioxidant defences.

Previous studies have reported that medicinal plants have compounds, such as ferulic acid can inhibit enzymes that catalyse free radical generation, while concurrently enhancing scavenger enzyme activity [52,74]. The observed antioxidant activity in HaCaT cells after exposure to the plant extracts could also be attributed to rutin, a potent antioxidant found in both *G. africana* and *T. violacea*. A previous study by Lang and Han [75] corroborates these results by showing that rutin can inhibit HaCaT cell oxidative stress by modulating the nuclear factor erythroid 2-related factor 2 (Nrf2), an important factor in the endogenous antioxidant defence system. The ability of these extracts to reduce intracellular ROS highlights their potential as protective agents against oxidative-induced cellular damage. The wound healing activities demonstrated by *G. africana* and *T. violacea* are likely supported by their antioxidant effects, which reduce oxidative stress at the site of injury and promote cellular repair.

## 4. Conclusions

The results of the bioactive assays conducted in the present study provide scientific validation for the traditional use of *G. africana* and *T. violacea* as wound healing agents, as both extracts demonstrated no cytotoxic effects and enhanced epidermal cell migration. *G. africana* demonstrated greater phenolic content and antioxidant activity, while *T. violacea* showed better wound migration. This study provides scientific evidence that supports the traditional use of *G. africana* and *T. violacea,* suggesting that these plants could serve as alternative agents that accelerate wound healing. HPLC analysis identified known phytoconstituents such as ferulic acid and coumaric acid in the plant extracts, all of which are known to possess antioxidant, anti-inflammatory and wound-healing properties. Future studies will evaluate the biological activity of these key compounds at their detected concentrations to determine their individual contributions to the wound healing effects observed in vitro. This will provide insight into the active constituents responsible for the bioactivity and form the basis for standardisation of the extract in future therapeutic applications. Additional research will include a broader, non-targeted phytochemical analysis using Liquid Chromatography–Mass Spectrometry (LC–MS) or Gas Chromatography–Mass Spectrometry (GC–MS) to identify other active compounds contributing to the plants’ wound-healing activity. Future studies will investigate the molecular mechanisms of the extracts, focusing on their specific signalling pathways and key protein markers. This research is essential to fully validate their safety, efficacy and potential as wound-healing agents.

## Figures and Tables

**Table 1:** Phytochemical composition and antioxidant activity of *Galenia africana* and *Tulbaghia violacea* extracts.

Treatment (1 mg/mL)	*G. africana* Ethanolic Extract	*T. violacea* Ethanolic Extract
Polyphenols (mg GAE/g)	106.39 ± 2.03	78.57 ± 2.40
Flavonoid content (mg QE/g)	39.79 ± 0.27	-
DPPH μmol TE/g	187.42 ± 6.40	117.60 ± 6.33
ORAC μmol TE/g	1247.83 ± 12.53	172.82 ± 12.39
FRAP μmol AAE/g	195.91 ± 4.26	116.25 ± 12.57

Abbreviations: TPC, total phenolic content; TFC, total flavonoid content; GAE, gallic acid equivalents; CE, catechin equivalents; AAE, ascorbic acid equivalents; QE, quercetin equivalent; DPPH,2,2-diphenyl-1-picrylhydrazyl. Values represent mean ± standard error of mean (n = 3).

**Table 2:** Identification and quantification of key bioactive compounds in *Galenia africana* and *Tulbaghia violacea* ethanolic extracts.

Plant Extract	Phytochemicals	Quantity (mg/L)	Retention Time (min)	Wavelength (nm)
*G. africana*	Ferulic acid	1.88	22.227	320
	Rutin	35.87	25.313	350
*T. violacea*	Rutin	1.18	24.003	350
	Coumaric acid	13.58	21.008	320
	Ferulic acid	1.88	22.185	320

## Data Availability

No new research data were created. All data was retrieved from published articles available to the public from various databases.
